# Comorbidities Associated with One-Year Mortality in Patients with Atrial Fibrillation and Heart Failure

**DOI:** 10.3390/healthcare9070830

**Published:** 2021-07-01

**Authors:** Ruxandra Nicoleta Horodinschi, Camelia Cristina Diaconu

**Affiliations:** 1 Department 5, “Carol Davila” University of Medicine and Pharmacy, 050474 Bucharest, Romania; ruxy691@yahoo.com; 2 Department of Internal Medicine, Clinical Emergency Hospital of Bucharest, 014461 Bucharest, Romania

**Keywords:** heart failure, atrial fibrillation, left ventricular ejection fraction, mortality

## Abstract

Background: Heart failure (HF) and atrial fibrillation (AF) commonly coexist and patients with both diseases have a worse prognosis than those with HF or AF alone. The objective of our study was to identify the factors associated with one-year mortality in patients with HF and AF, depending on the left ventricular ejection fraction (LVEF). Methods: We included 727 patients with HF and AF consecutively admitted in a clinical emergency hospital between January 2018 and December 2019. The inclusion criteria were age of more than 18 years, diagnosis of chronic HF and AF (paroxysmal, persistent, permanent), and signed informed consent. The exclusion criteria were the absence of echocardiographic data, a suboptimal ultrasound view, and other cardiac rhythms than AF. The patients were divided into 3 groups: group 1 (337 patients with AF and HF with reduced ejection fraction (HFrEF)), group 2 (112 patients with AF and HF with mid-range ejection fraction (HFmrEF)), and group 3 (278 patients with AF and HF with preserved ejection fraction (HFpEF)). Results: The one-year mortality rates were 36.49% in group 1, 27.67% in group 2, and 27.69% in group 3. The factors that increased one-year mortality were chronic kidney disease (OR 2.35, 95% CI 1.45–3.83), coronary artery disease (OR 1.67, 95% CI 1.06–2.62), and diabetes (OR 1.66, 95% CI 1.05–2.67) in patients with HFrEF; and hypertension in patients with HFpEF (OR 2.45, 95% CI 1.36–4.39). Conclusions: One-year mortality in patients with HF and AF is influenced by different factors, depending on the LVEF.

## 1. Introduction

Heart failure (HF) and atrial fibrillation (AF) are common cardiac diseases, which are frequently related and share common risk factors, such as old age, hypertension (HT), coronary artery disease (CAD), valvular heart disease, diabetes mellitus (DM), and chronic kidney disease (CKD) [1,2,3,4,5]. HF is a clinical syndrome caused by structural or functional cardiac abnormalities leading to diminished cardiac output, elevated intracardiac pressure, or both [1]. According to the 2021 European Society of Cardiology Guidelines for the management of patients with HF, there are three types of HF: HF with reduced ejection fraction (HFrEF)—left ventricular ejection fraction (LVEF) < 40%; HF with mid-range ejection fraction (HFmrEF)—LVEF in the range of 40–49%; HF with preserved ejection fraction (HFpEF)—LVEF ≥ 50% [1]. The diagnosis of HF is based on the presence of clinical signs and symptoms of heart failure, elevated levels of natriuretic peptides, and at least one additional criteria of relevant structural heart disease (left ventricular hypertrophy or left atrial enlargement) or diastolic dysfunction [1]. The New York Heart Association (NYHA) classification provides a simple way to characterize the symptoms of HF, as follows: NYHA class I—no limitation of physical exercise; NYHA class II—mild limitation of physical exercise, ordinary physical activity leads to fatigue, dyspnea, but comfortable at rest; NYHA class III—important limitation of physical activity, less than ordinary activity leads to fatigue, dyspnea, comfortable at rest; NYHA class IV—dyspnea at rest, unable to do any physical exercise without discomfort [1].

The management of patients with HF differs according to their LVEF [1]. Patients with HFrEF benefit from specific treatments that reduce mortality, such as beta blockers, angiotensin-converting enzyme inhibitors, angiotensin receptor blockers, angiotensin receptor neprilysin inhibitors (ARNI), sodium–glucose cotransporter 2 (SGLT-2) inhibitors, and mineralocorticoid antagonists especially eplerenone, while in patients with HFpEF none of these drugs have proved to reduce mortality; thus, in patients with HFpEF, the management is based mainly on symptomatic therapy and treatment of comorbidities.

AF is the most common sustained cardiac arrhythmia, with important impacts on morbidity, mortality, and quality of life [6,7,8,9,10]. Several studies have reported that AF increases all-cause and cardiovascular mortality [11,12]. It may lead to thromboembolic events, such as stroke or transient ischemic attack [13]. The prevalence of both AF and HF is increasing, especially with the aging of the population [14,15,16]. The association of HF and AF is quite common. For example, the Acute Decompensated Heart Failure National Registry (ADHERE) evaluated the medical records of 107,362 patients hospitalized for HF decompensation, of whom 31% had a history of AF [17]. Patients with HF and AF have a worse prognosis than those with each disease alone [18,19,20,21,22]. Each one of these two diseases can lead to the development and worsening of the other. AF can favor HF progression through tachycardia-related cardiomyopathy characterized by the loss of atrial systole, left atrial dilation, and a rapid and irregular ventricular rate that affects left ventricular emptying and filling [23]. HF may induce AF through increased left ventricular stiffness, elevated LV filling pressure, and neurohormonal activation [24]. The presence of both conditions leads to a higher risk of stroke, a more important deterioration of the cardiac function, and more severe symptoms [1,24]. HF and AF are both associated with significant morbidity and mortality [15]. Patients with HF and AF have a 14% to 57% higher risk of death than those with HF in sinus rhythm [25,26]. A study by Fauchier et al. included 8962 patients with AF and revealed that one of the three main causes of death in the cohort was HF, independently of the LVEF [10].

The objective of the study was to identify the risk of one-year mortality patients with chronic HF and AF and to determine the comorbidities that increase one-year mortality, according to LVEF. The risk of one-year mortality after discharge was chosen because this timeframe is critical for the response to recommended treatment and for assessing the impacts of comorbidities on patient prognosis.

## 2. Materials and Methods

### 2.1. Study Population

This was a prospective, observational case–control study. We recruited 2878 consecutively admitted patients with the diagnosis of HF, hospitalized in the Clinical Emergency Hospital of Bucharest, Romania, between January 2018 and December 2019. Of these patients, we selected 942 patients with AF (paroxysmal, persistent, long-standing persistent, or permanent).

The inclusion criteria were age of more than 18 years, diagnosis of HF and AF, and signed informed consent to participate in this study.

The exclusion criteria were the absence of echocardiographic data, a suboptimal ultrasound view leading to the imposibility to obtain minimum echocardiographic data (at least the LVEF), and other cardiac rhythms than AF.

After applying the inclusion and exclusion criteria, 727 patients with AF and HF remained in the study. The diagnosis of HF was established based on the European Society of Cardiology Guidelines [1].

The diagnosis of AF was made using a standard 12-lead electrocardiogram or automated continuous monitoring of cardiac rhythm for 24–72 h.

**Figure 1:**
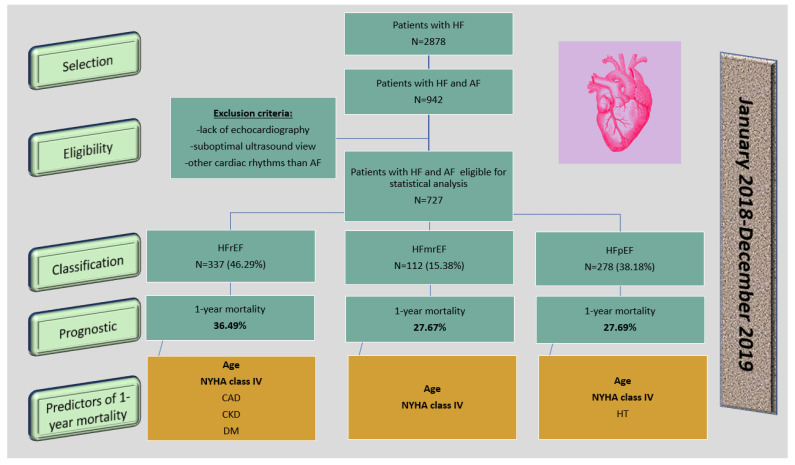
The protocol of the study. Legend: HF—heart failure; AF—atrial fibrillation; HFrEF—heart failure with reduced ejection fraction; HFmrEF—heart failure with mid-range ejection fraction; HFpEF—heart failure with preserved ejection fraction; NYHA—New York Heart Association; CAD—coronary artery disease; CKD—chronic kidney disease; DM—diabetes mellitus; HT—hypertension.

The patients were divided into three subgroups according to the LVEF: subgroup 1 included 337 patients with HFrEF (46.29%), subgroup 2 included 112 patients with HFmrEF (15.38%), and subgroup 3 included 278 patients with HFpEF (38.18%). The study protocol is shown in Figure 1.

Baseline demographics were obtained at inclusion in the study. We collected the following data about the patients: general and demographic data, medical history, current disease status, reason for hospitalization, current therapy, and comorbidities. The diagnosis of the comorbidities was established at inclusion in the study. Valvular pathologies were assessed by echocardiography and only patients with moderate or severe valvular disease were included in the study. The diagnosis of CAD was established by non-invasive tests for myocardial ischemia, such as stress echocardiography, stress myocardial scintigraphy, cardiac magnetic resonance imaging, or by invasive assessment by angiography. Patients with all degrees of myocardial ischemia were included. These included enrolled patients with a prior diagnosis of CKD and with a glomerular filtration rate < 60 mL/min/1.73 m^2^, with a prior diagnosis of DM, or with newly diagnosed DM.

The study respected the ethical standards of the Helsinki Declaration of 1975, as revised in 2008(5), as well as the national law. We ensured that patients’ rights were protected and the confidentiality of their data was maintained. The study was approved by the Ethics Committee of the Clinical Emergency Hospital of Bucharest, Romania (approval number 4714/24.05.2019).

### 2.2. Laboratory Tests

In order to completely assess the comorbidities of patients with HF and AF, such as CAD, CKD, DM, dyslipidemia, and liver diseases, blood tests were performed on all patients enrolled in the study using an ABX pentra XL 80 hematology autoanalyzer for complete cell blood counts (leukocytes 4000–9000/µL; hemoglobin 12.6–17.2 g/dL; platelets 150.000–350.000/µL) and a BioMajesty biochemistry autoanalyzer for creatinine (0.7–1.4 mg/dL), blood urea nitrogen (19–43 mg/dL), serum sodium (137–145 mmol/L), serum potassium (3.5–5 mmol/L), aminotransferases (aspartate aminotransferase 14–50 U/L; alanine aminotransferase 10–50 U/L), glycemia (75–110 mg/dL), troponin I (<5 ng/mL), creatine kinase (55–170 U/L), creatine kinase-MB (10–16 U/L), total cholesterol (140–200 mg/dL), and triglycerides (30–150 mg/dL).

### 2.3. Echocardiography

In this study, 2D transthoracic echocardiography was performed on all patients enrolled in the study, either at inclusion in the study or the data were extracted from the medical records of the patients if the data were consistent with our examination protocol.

We used commercially available ultrasound systems, such as the Phillips CX 50 or Vivid 9 machine. Conventional measures, such as the dimensions of the LV walls, LV end-diastolic and end-systolic diameters and volumes, left atrial diameter and volume, right atrial diameter and area, and right ventricular diameter were obtained. LVEF was calculated in the apical 4- and 2-views using the modified Simpson’s biplane method, or if this was not possible, LVEF was visually estimated. Valvular pathologies were evaluated using color, pulse, and continuous Doppler measurements. The aorta and pericardium were also assessed.

### 2.4. Statistical Analysis

Statistical analysis was performed using R software version 4.0.2 (c) R Core Team 2020 (R: a language and environment for statistical computing; R Foundation for Statistical Computing, Austria). Descriptive statistics are presented as absolute frequencies, mean values ± standard deviation, and medians with interquartile range. In order to identify the factors that may influence the mortality in the group of study, a binomial univariate logistic regression was used. At first, a simple logistic regression was used with a single predictor rather than multiple logistic regression, with a backward selection algorithm for multiple predictors of mortality. The dependent variable (“output”) was the rate of mortality and the independent variables (“input”) were the individual clinical (symptoms of HF that were classified according to NYHA classification, signs of HF) and demographic (age, sex) characteristics and the comorbidities of the patients. Normally distributed data are expressed as means ±standard deviation. Data deviating from the normal range are expressed as median values. The analysis of variance (ANOVA) was applied to compare the differences among the mean ages of the three groups of patients. The chi-squared test (χ^2^ test) was used to estimate whether there were statistically significant differences between the frequencies of several characteristics and comorbidities of the patients in the three groups. A value of *p* < 0.05 was considered to be statistically significant.

## 3. Results

Baseline characteristics of patients with HF and AF, according to their LVEF, are shown in Table 1. Almost half of the patients (46.35%) had HFrEF, 38.23% had HFpEF, and 15.4% had HFmrEF.

Patients with HFpEF were significantly older (mean age 76.16 ± 9.58 years) than those with HFrEF (mean age 70.77 ± 11.15 years). The proportion of females was greater compared to males (60.08% versus 39.92%) in the group of patients with HFpEF. In the group of patients with HFrEF, there were more men than women (68.84% versus 31.16%).

The rate of one-year mortality for patients with HF and AF depending on their LVEF was 27.69% in patients with HFpEF, 27.67% in those with HFmrEF, and 36.49% in HFrEF.

Firstly, a simple binomial regression model was performed to identify the one-year mortality predictors for every subgroup of patients. HT was associated with increased one-year mortality in patients with HFpEF (OR 2.45, 95% CI 1.36 to 4.39) (Table 2). Moreover, in patients with HFpEF, age was directly linked to the rate of death. Consequently, a one-year increase in age led to a 10% higher risk of one-year mortality.

In patients with HFrEF, we determined the following factors associated with increased mortality: CAD–OR 1.67, 95% CI 1.06 to 2.62; aortic regurgitation (AR)–OR 1.95, 95% CI 1.09 to 3.50; aortic stenosis (AS)–OR 2.00, 95% CI 1.04 to 3.83; CKD–OR 2.35, 95% CI 1.45 to 3.83; DM–OR 1.66, 95% CI 1.05 to 2.67 (Table 2). In these patients, a one-year increase in age was associated with a 4% higher risk of mortality.

NYHA class IV was associated with higher risk of mortality in all three groups of patients compared to NYHA class II, as follows: in patients with HFpEF, NYHA class IV had a 2.45-fold higher OR than NYHA class II; in patients with HFmrEF, NYHA class IV had a 4-fold higher OR than NYHA class II; in patients with HFrEF, NYHA class IV had a 3.86-fold higher OR than NYHA class II. In patients with HFrEF, NYHA class III was also associated with a greater risk of mortality compared to NYHA class II (2.25-fold increase of OR).

Uncommon comorbidities in the study group, such as mitral stenosis, chronic obstructive pulmonary disease, and sleep apnoea syndrome, were mentioned in the general description of the patients (Table 2) but were not included in the statistical analysis.

A multiple univariate binomial logistic regression model was performed in patients with HFpEF, revealing that the independent predictors for one-year mortality were age (one-year increase in age was associated with a 9% higher OR), NYHA class IV (3-fold higher OR than NYHA class I/II), and HT (2.55-fold OR compared to normal blood pressure) (Table 3); thus, in patients with HFpEF, both simple and multiple regression models identified the same predictors for one-year mortality, which were advanced age, NYHA class IV, and HT.

A multiple univariate binomial logistic regression model was also performed in patients with HFrEF, using all statistically significant predictors that influenced mortality in the simple model in order to identify the independent predictors. Not all predictors of mortality from the simple regression model appeared as independent predictors in the multiple regression model, such as reduced LVEF, CAD, and DM (Table 4); therefore, independent predictors of mortality in patients with HFrEF were age (one-year increase in age was associated with a 3% higher OR), NYHA class III (2.24-fold higher OR than NYHA class I/II), NYHA class IV (3.79-fold higher OR than NYHA class I/II), and CKD (2-fold higher OR) (Table 4).

## 4. Discussion

The objective of this study was to identify the characteristics and comorbidities that influence the one-year mortality in patients with HF and AF according to their LVEF and the differences between the three subgroups of patients.

Advanced age represents a risk factor for mortality in patients with HFrEF and HFpEF. Patients with severe symptoms, namely NYHA class III or IV, had a higher risk of mortality than those with mild symptoms, regardless of their LVEF. Patients with HFpEF were older than those with HFrEF. Sex differences were noticed between the subgroups; therefore, patients with HFpEF were more frequently women, while those with HFrEF were more frequently men. The predominance of women among patients with HFpEF represents one of the most important differences between patients with HFpEF and those with HFrEF. Women have higher myocardial stiffness, leading to increased LV filling pressures and diastolic dysfunction but greater LVEF compared to males [27,28]. Women have higher pulmonary capillary wedge pressure indexed to peak exercise workload and lower systemic and pulmonary arterial compliance during exercise compared to men, leading to higher LV filling pressures, as measured by echocardiographic or invasive methods [29]. Women seem to have more severe symptoms of HF, greater functional impairement, and worse quality of life but lower risk of hospitalization for HF decompensation and all-cause and cardiovascular death than men [30,31].

In the current study, patients with HFrEF had a higher risk of one-year mortality compared to those with HFmrEF or HFpEF. The risk of mortality was similar in patients with HFpEF and HFmrEF. The risks of mortality in patients with AF and HF have been discussed in several clinical trials, including patients with all types of HF [23,32,33]. The majority of the studies concluded that the association of the two conditions leads to the increase of mortality risk compared to the individual risk of each disease alone [23,32,33]. For example, Zhirov et al. enrolled 1003 patients with HF and AF and concluded that mortality increased proportionally with the decrease of the LVEF by 4.1% in patients with HFpEF, 9.3% in patients with HFmrEF, and 15.5% in patients with HFrEF [32]. Crijins et al. enrolled 409 patients who had previously participated in the Prospective Randomized study of Ibopamine on Mortality and Efficacy (PRIME-II), of whom 325 patients were in sinus rhythm and 84 patients had AF, and demonstrated that patients with AF and HF have a worse prognosis than those in sinus rhythm, with a higher mortality rate [33]. The Candesartan in Heart Failure—Assessment of Reduction in Mortality and Morbidity (CHARM) trial included 7599 patients with chronic HF who were randomized to candesartan or placebo and concluded that AF is associated with an elevated risk of mortality and morbidity in all patients with HF, especially in those with LVEF > 40% [23]. Son et al. evaluated 5414 patients with HF, of whom 1883 patients (34.8%) had AF [34]. AF was more frequent in patients with HFpEF and patients with HFpEF and AF had a higher risk of mortality than those with AF and HFrEF [34].

Our study focused on determining if there are different factors and comorbidities influencing mortality in the three subgroups of patients. In patients with HFpEF and AF, HT was a risk factor that increased one-year mortality. HT is a well-known cardiovascular risk factor and is the most common comorbidity in patients with HFpEF [35]. Systemic HT increases LV afterload, leading to LV hypertrophy and diastolic dysfunction. In the hypertrophic myocytes, there is diminished capillary density, limited vasodilation of the coronary arteries, and consequently an elevated risk of myocardial ishemia. HT leads to a systemic proinflammatory state that may cause coronary microvascular endothelial dysfunction and reduced levels of nitric oxide and protein kinase C activity [36]. Reduced activity of protein kinase C leads to myocardium hypertrophy. All these mechanisms favor increased stiffness and high filling pressures of the LV and consequently diastolic dysfunction [36]. Patients with HFpEF do not benefit from a specific therapy in terms of decreasing mortality, in contrast to those with HFrEF; in these patients, the therapeutic management is focused on the treatment of their comorbidities. Antihypertensive treatment is an important point in the management of patients with HFpEF, which may lead to diastolic function improvement [33].

In the current study, CAD, CKD, and DM increased the risk of death in the subgroup of patients with HFrEF and AF. Myocardial ischemia, especially if prolonged or recurrent, leads to LV remodelling, dilation, and systolic dysfunction. After myocardial infarction, a proinflammatory reaction appears as a response to cardiomyocyte injury in order to mediate tissue repair; however, an excessive inflammatory response may induce myocardial injury; thus, the degree of the inflammatory reaction seems to be an important point in HF development after myocardial infarction [37]. Increased C-reactive protein levels in the first 24–48 h after acute myocardial infarction may be a predictor for long-term HF development and mortality [37]. Patients who develop HF after myocardial infarction have higher risks of recurrent infarction and mortality than those without HF [37].

The therapeutic options for patients with CAD include medical, interventional, or surgical treatment. Several studies have evaluated the efficiency of these therapeutic methods regarding the prognosis of patients with HFrEF and CAD [38,39,40]. Myocardial revascularization is superior to drug treatment in improving survival in patients with HF of ischemic cause [41]. The optimal revascularization strategy varies depending on the type of CAD (chronic or acute), the severity of the coronary lesion (obstructive or non-obstructive), and the number of injured vessels. In patients with acute coronary syndrome, urgent coronary angiography and angioplasty of the responsible artery are recommended [41]. In patients with HFrEF and chronic CAD of ischemic origin, myocardial revascularization is recommended [42,43]. Coronary artery bypass grafting is the first choice in patients with multivessel disease and acceptable surgical risk, especially in those with three-vessel disease [41]. In patients with one- or two-vessel disease, percutaneous coronary intervention may be taken into account when complete revascularization is possible [41]. Mortality is significantly lower in patients with ischemic HFrEF treated by coronary artery bypass grafting than in those receiving medical treatment [38,43]. An implantable cardioverter–defibrillator (ICD) may be recommended in patients with HFrEF for primary or secondary prevention of sudden cardiac death. ICD can prevent bradycardia and correct ventricular arrhythmias, which may be lethal. For primary prevention, ICD is recommended in patients with asymptomatic left ventricular systolic dysfunction (LVEF ≤ 30%) of ischemic cause who are at least 40 days after an acute myocardial infarction, in patients with non-ischemic dilated cardiomyopathy with LVEF ≤ 30% who receive optimal medical treatment, in patients with class II-III NYHA symptoms and LVEF ≤ 35% in spite of optimal medical treatment for at least three months to improve survival [39]. For secondary prevention, ICD is recommended in patients who have survived a ventricular arrhythmia complicated by hemodynamic instability in order to reduce the risk of sudden cardiac death [39].

The results of the current study showed that patients with HFrEF, AF, and CKD had a higher risk of mortality than those with HFrEF, AF, and normal renal function. HF and CKD are chronic disorders that frequently coexist [44]. Their incidence and prevalence increase with age [45]. Each one of these two diseases seems to predispose sufferers to the progression of the other, and their association increases the risk of hospitalization and mortality [46]. A decrease of the glomerular filtration rate may lead to increases of hospitalization and all-cause and cardiovascular death in patients with both HFrEF and HFpEF [47,48]. CKD contributes to HF through uncontrolled blood pressure and water and salt retention, leading to increased preload and excessive arterial stiffness, causing increased afterload, neurohormonal activation, anemia, and excess of profibrotic factors such as fibroblast growth factor 23 [49]. All of these factors favor LV hypertrophy and fibrosis; therefore, myocardial hypertrophy leads to low capillary density and ischemia through the imbalance between oxygen demand and supply, cardiomyocyte apoptosis, and extracellular collagen deposition, with fibrosis and LV stiffness [50]. Myocardial fibrosis worsens ischemia by reducing coronary reserve and increases the risk of ventricular arrhythmias and sudden cardiac death [51,52,53]. At first, LV hypertrophy appears as an adaptative remodelling process as a result of increased preload due to hypervolemia, high blood flow through the arteriovenous fistula in hemodyalisis patients, and afterload due to hypertension and arterial stiffness. Except for hemodynamic factors that lead to LV hypertrophy, in CKD there are particular factors that predispose sufferers to LV hypertrophy, including hyperphosphatemia, which may cause increased LV mass and arterial hypertension; activation of the renin–angiotensin–aldosterone system, causing increased myocardial fibrosis; and increased activity of sympathetic system [50]. According to Lofman et al., the presence of CKD in patients with HF may lead to increased rate of mortality independently of age, increased duration of HF, or increased functional class of HF [54]. Another factor that may influence the prognosis of patients with HFrEF and concomitant CKD, especially in those with glomerular filtration rate < 30 mL/min/1.73 m^2^, is that these patients are less likely to receive the specific therapies that reduce mortality in HFrEF, such as angiotensin-converting enzyme inhibitors, angiotensin receptor blockers, ARNI, SGLT-2 inhibitors, and mineralocorticoid antagonists, because of kidney dysfunction and hyperkalemia. Patients with HFrEF and glomerular filtration rates < 30 mL/min/1.73 m^2^ have usually been excluded from clinical trials that have evaluated the effects of the drugs mentioned above because of their possible toxicity in these patients; therefore, patients with HFrEF and advanced CKD represent a special category of patients with fewer therapeutic options and a worse prognosis.

DM is another factor that increased the risk of mortality in patients with HFrEF and AF enrolled in this study. DM is a frequent comorbidity in patients with HF, which leads to a higher risk of death in patients with both HFrEF and HFpEF compared to patients without DM [55,56,57,58]. Impaired systemic and cardiac glucose metabolism in patients with DM may lead to HF through several pathophysiological and metabolic disorders, such as excessive oxidation of free fatty acids, which may decrease myocardial contractility, inducing systolic dysfunction even in the absence of CAD or structural heart disease [55]. HF in diabetic patients may also be caused by decreased transport of glucose and sarcolemal calcium in the cardiomyocytes, low carbohydrates oxidation, and dysfunction of the myofibrillar contractile proteins [55]. The concentrations of glucose transporters one and four are diminished in patients with DM, causing slow glucose transport through the sarcolemal membrane and uptake in the cardiomyocytes [55]. Increased free fatty acid oxidation inhibits glycolysis and glucose oxidation in the myocardium [55]; therefore, adenosine triphosphate is obtained from free fatty acid oxidation, not from glucose as in patients without DM, leading to low cardiac energy reserves and HF [55]. Hyperglycemia and insulin resistance lead to microvascular endothelial dysfunction, impaired cardiac metabolism, increased myocardial fibrosis, oxidative stress, and activation of the renin–angiotensin system and sympathetic nervous system [59]. In the CHARM trial, the association of insulin-requiring DM in patients with HF led to a 2-fold higher risk of mortality (general and cardiovascular) and hospitalization for HF [60]. DM therapy represents an important point in the management of patients with HF, although according to several studies, intensive glucose-lowering therapy leading to strict control of glycemia and glycated hemoglobin seems to have limited advantages in reducing mortality in patients with HF and DM [61,62,63].

Our results showed that advanced age, advanced functional class of HF, and reduced LVEF increased the one-year mortality in patients with HF and AF. We demonstrated that there are different comorbidities that influence one-year mortality in patients with HF and AF, depending on the LVEF, such as HT in patients with HFpEF and CAD, CKD, and DM in patients with HFrEF. All these diseases aggravate each other and their association leads to a significantly worse prognosis and reduced survival.

The main limitation of our study is the relatively low number of patients with HFmrEF included, which is fewer than those with HFpEF and HFrEF; this could explain why we have not found any particular factors associated with one-year mortality in this group of patients, except for age and functional class of HF. There were also possible differences in appreciating the ultrasound parameters because of the different examiners who performed transthoracic echocardiography.

Future research may be focused on evaluating whether an early diagnosis and treatment of the comorbidities in patients with HF and AF may influence the prognosis of these patients with multiple pathologies by reducing the mortality. Another area of research may be focused on evaluating the benefits of the therapeutic strategy of CAD, namely interventional (angioplasty) versus surgical (coronary artery bypass grafting) options, in patients with HF and AF. Another point of interest for research may be to determine whether the duration of AF influences the one-year mortality in patients with HF. Research may be also focused on determining how the severity levels of CAD (stable CAD or acute coronary syndrome), CKD (patients on dialysis or not), and DM (treated with oral antidiabetics or insulin-requiring) may influence mortality in each of the subgroups of patients with HF and AF.

## 5. Conclusions

HF and AF are some of the most common cardiovascular disorders, which frequently coexist. The association of these two pathologies leads to an important rate of mortality, especially in patients with HFrEF. This study demonstrated whether advanced age and functional class of HF are predictors of mortality in all patients with HF, regardless of the LVEF. Furthermore, we determined that one-year mortality is influenced by different factors, such as HT in patients with HFpEF and CAD, CKD, and DM in patients with HFrEF.

## Figures and Tables

**Table 1:** Baseline characteristics of patients with HF and AF depending on their LVEF.

Variable		HFpEF (N = 278)	HFmrEF (N = 112)	HFrEF (N = 337)	p-Value
Age-Mean ± SD		76.16 ± 9.58	72.54 ± 9.71	70.77 ± 11.15	<0.0001 ^1^
Sex M F		111/278 (39.92)	66/112 (58.92)	232/337 (68.84)	<0.0001 ^2^
		167/278 (60.08)	46/112 (41.08)	105/337 (31.16)	
NYHA	I/II III IV	128/278 (46.04)	37/112 (33.03)	57/337 (16.91)	<0.0001 ^2^
		82/278 (29.49)	34/112 (30.35)	105/337 (31.57)	
		68/278 (24.46)	41/112 (36.60)	175/337 (51.92)	
CAD		92/278 (33.09)	50/112 (44.64)	170/337 (50.44)	<0.0001 ^2^
MR		155/278 (55.75)	60/112 (53.57)	219/337 (64.98)	0.0240 ^2^
MS		13/278 (4.67)	3/112 (2.67)	8/337 (2.43)	0.2600 ^2^
AR		69/278 (24.82)	26/112 (23.21)	56/337 (16.61)	0.0349 ^2^
AS		61/278 (21.94)	15/112 (13.39)	43/337 (12.75)	0.0059 ^2^
TR		81/278 (29.13)	40/112 (35.71)	127/337 (37.68)	0.0779 ^2^
HT		212/278 (76.25)	74/112 (66.07)	211/337 (62.61)	0.0012 ^2^
CKD		91/278 (32.73)	28/112 (25.00)	96/337 (28.48)	0.2660 ^2^
DM		95/278 (34.17)	40/112 (35.71)	112/337 (33.23)	0.8880 ^2^
COPD		17/278 (6.11)	14/112 (12.50)	29/337 (8.60)	0.1110 ^2^

^1^ ANOVA. ^2^ χ^2^ test between groups. Legend: SD—standard deviation; HFpEF—heart failure with preserved ejection fraction; HFmrEF—heart failure with mid-range ejection fraction; HFrEF—heart failure with reduced ejection fraction; NYHA—New York Heart Association; CAD—coronary artery disease; MR—mitral regurgitation; MS—mitral stenosis; AR—aortic regurgitation; AS—aortic stenosis; TR—tricuspid regurgitation; HT—hypertension; CKD—chronic kidney disease; DM—diabetes mellitus; COPD—chronic obstructive pulmonary disease; ANOVA—analysis of variance; χ^2^ test—chi-square test.

**Table 2:** Predictors of one-year mortality in patients with HF and AF, according to the LVEF.

		HFpEF		HFmrEF		HFrEF	
Predictors		*p*-Value	or [95% CI]	*p*-Value	OR [95% CI]	*p*-Value	OR [95% CI]
Age		<0.0001	1.10 [1.06 to 1.14]	0.0546	1.04 [1.00 to 1.09]	0.0001	1.04 [1.02 to 1.06]
Sex M vs. F		0.1950	0.69 [0.39 to 1.19]	0.1631	0.55 [0.23 to 1.27]	0.5130	0.85 [0.53 to 1.37]
NYHA	I/II III IV	REF 0.0634 <0.0001	- 1.87 [0.96 to 3.67] 4.28 [2.22 to 8.40]	0.1082 0.0146	- 2.66 [0.83 to 9.52] 4.09 [0.86 to 13.93]	- 0.0456 0.0003	- 2.25 [1.04 to 5.20] 3.86 [1.90 to 8.55]
CAD		0.4800	0.81 [0.45 to 1.42]	0.7215	0.85 [0.36 to 1.97]	0.0248	1.67 [1.06 to 2.62]
MR		0.5770	1.16 [0.68 to 1.98]	0.5558	1.28 [0.55 to 3.01]	0.8250	0.94 [0.59 to 1.51]
MS		0.8000	1.16 [0.30 to 3.70]	0.3600	0.24 [0.10 to 1.58]	0.9530	1.04 [0.21 to 4.33]
AR		0.0694	1.71 [0.95 to 3.96]	0.9250	0.95 [0.33 to 2.47]	0.0230	1.95 [1.09 to 3.50]
AS		0.3160	1.36 [0.73 to 2.50]	0.0994	2.45 [0.85 to 8.21]	0.0348	2.00 [1.04 to 3.83]
TR		0.4500	1.24 [0.69 to 2.18]	0.6467	0.81 [0.32 to 1.91]	0.2157	0.74 [0.46 to 1.17]
HT		0.0025	2.45 [1.36 to 4.39]	0.8226	0.90 [0.36 to 2.14]	0.6410	1.11 [0.70 to 1.76]
CKD		0.2790	1.35 [0.77 to 2.33]	0.1290	1.98 [0.82 to 5.13]	0.0005	2.35 [1.45 to 3.83]
DM		0.7110	0.89 [0.50 to 1.56]	0.2120	1.69 [0.74 to 4.07]	0.0305	1.66 [1.05 to 2.67]
COPD		0.2060	1.91 [0.67 to 5.16]	0.9400	1.04 [0.27 to 3.44]	0.0712	0.42 [0.15 to 1.01]

Legend: HFpEF—heart failure with preserved ejection fraction; HFmrEF—heart failure with mid-range ejection fraction; HFrEF—heart failure with reduced ejection fraction; OR—odds ratio; CI—confidence interval; NYHA—New York Heart Association; CAD—coronary artery disease; MR—mitral regurgitation; MS—mitral stenosis; AR—aortic regurgitation; AS—aortic stenosis; TR—tricuspid regurgitation; HT—hypertension; CKD—chronic kidney disease; DM—diabetes mellitus; COPD—chronic obstructive pulmonary disease.

**Table 3:** Independent redictors of mortality in patients with HFpEF.

Predictor		*p*-Value	OR [95% CI]
Age		<0.0001	1.09 [1.05 to 1.13]
NYHA	I/II III IV	REFERENCE 0.2776 0.0025	- 1.48 [0.72 to 3.04] 3.00 [1.47 to 6.19]
HT		0.0046	2.55 [1.33 to 4.90]

Legend: OR—odds ratio; CI—confidence interval; NYHA—New York Heart Association; HT—hypertension.

**Table 4:** Independent predictors of mortality in patients with HFrEF.

Predictor		*p*-Value	OR [95% CI]
Age		0.0015	1.03 [1.01 to 1.06]
NYHA	I/II III IV	REFERENCE 0.0500 0.0005	- 2.24 [1.02 to 5.28] 3.79 [1.83 to 8.53]
CKD		0.0065	2.02 [1.21 to 3.38]

Legend: OR—odds ratio; CI—confidence interval; NYHA—New York Heart Association; CKD—chronic kidney disease.
